# Dataset on equine cartilage near infrared spectra, composition, and functional properties

**DOI:** 10.1038/s41597-019-0170-y

**Published:** 2019-08-30

**Authors:** Jaakko K. Sarin, Jari Torniainen, Mithilesh Prakash, Lassi Rieppo, Isaac O. Afara, Juha Töyräs

**Affiliations:** 1 Department of Applied Physics, University of Eastern Finland, Kuopio, Finland; 2 Diagnostic Imaging Center, Kuopio University Hospital, Kuopio, Finland; 3 A.I. Virtanen Institute for Molecular Sciences, University of Eastern Finland, Kuopio, Finland; 4 Research Unit of Medical Imaging, Physics and Technology, Faculty of Medicine, University of Oulu, Oulu, Finland; 5 School of Information Technology and Electrical Engineering, The University of Queensland, Brisbane, Australia

**Keywords:** Biomedical engineering, Biophysics, Cartilage, Near-infrared spectroscopy

## Abstract

Near infrared (NIR) spectroscopy is a well-established technique that is widely employed in agriculture, chemometrics, and pharmaceutical engineering. Recently, the technique has shown potential in clinical orthopaedic applications, for example, assisting in the diagnosis of various knee-related diseases (*e*.*g*., osteoarthritis) and their pathologies. NIR spectroscopy (NIRS) could be especially useful for determining the integrity and condition of articular cartilage, as the current arthroscopic diagnostics is subjective and unreliable. In this work, we present an extensive dataset of NIRS measurements for evaluating the condition, mechanical properties, structure, and composition of equine articular cartilage. The dataset contains NIRS measurements from 869 different locations across the articular surfaces of five equine fetlock joints. A comprehensive library of reference values for each measurement location is also provided, including results from a mechanical indentation testing, digital densitometry imaging, polarized light microscopy, and Fourier transform infrared spectroscopy. The published data can either be used as a model of human cartilage or to advance equine veterinary research.

## Abstract (summary)

**Table:** 

Design Type(s)	biomechanics data analysis objective • methodology testing objective
Measurement Type(s)	cartilage • biomechanical data • histology
Technology Type(s)	near-infrared spectroscopy • biomechanical data analysis
Factor Type(s)	Region_Of_Interest • Biospecimen • metacarpophalangeal joint
Sample Characteristic(s)	Equus • metacarpophalangeal joint

Machine-accessible metadata file describing the reported data (ISA-Tab format)

## Background & Summary

Osteoarthritis (OA) is the most common musculoskeletal disorder^1^, characterized by the deterioration of articular cartilage and subchondral bone. Articular cartilage is an avascular and aneural connective tissue that covers the ends of bones in articulating joints (*e*.*g*., the knee). The main role of articular cartilage is to enable near-frictionless movement between the bones and to distribute mechanical loads. OA can be triggered by prolonged abnormal joint loading (for example, being overweight), whereas post-traumatic OA (PTOA) can be caused by sudden physical trauma, such as a fall or a slip^2–5^. Once triggered, the OA progresses due to the poor self-repair capabilities of articular cartilage. Currently, no cure exists for OA; however, the progression of the disease can be slowed down with medication and conventional physical therapy. Advanced OA is typically very painful and can eventually require joint replacement surgery^6^. Furthermore, OA-related medical costs and disabilities make it a substantial socio-economic burden. Since cartilage is an aneural tissue, early-stage OA can be asymptomatic and, therefore, difficult to detect. In order to effectively plan treatment and improve the outcomes of OA patients, early and accurate diagnosis of cartilage defects is vitally important.

Initial diagnosis of OA is usually performed by a medical doctor through physical examination. Modern medical imaging techniques are effective for verifying the initial detection of OA. However, during joint repair procedures, surgeons rely on traditional arthroscopic tools when evaluating cartilage condition. These tools are currently limited to an endoscopic camera (for visual observation) and a metal hook (for manual palpation of the tissue)^7^. While these methods are considered to be the gold standard, they are highly subjective with poor repeatability^8–10^. Quantitative techniques for real-time diagnostics of cartilage could substantially improve the decision-making ability of orthopaedic surgeons during these repair procedures^11–13^.

Near infrared spectroscopy (NIRS) has demonstrated potential as a diagnostic tool for evaluating cartilage condition^12,14,15^. NIRS is a non-destructive optical sensing technique in which the absorbance of a target sample at different wavelengths within the near infrared (NIR) spectral range is measured. Changes in the composition and structure of cartilage can be observed in the measured spectrum. As OA-related degeneration of articular cartilage induces changes in both the chemical composition and structure, NIRS can indirectly quantify the condition of the tissue.

NIRS-based arthroscopy requires sophisticated multivariate statistical models (such as, partial least squares regression, principal component regression, neural networks, etc.) that relate the measured NIR spectra to the various properties of cartilage. To develop any of these techniques, large data library from human cadavers or suitable animal models (*i*.*e*., large mammals) is often required. Ideally, these datasets should consist of multiple cartilage samples with varying degrees of tissue defects. In addition to NIRS measurements, a set of reference variables (*i*.*e*., different biomechanical and chemical properties that characterize the tissue) should be included. To facilitate the development of better NIRS models for cartilage evaluation, we are publishing this dataset collected from equine fetlock joints.

**Fig. 1:**
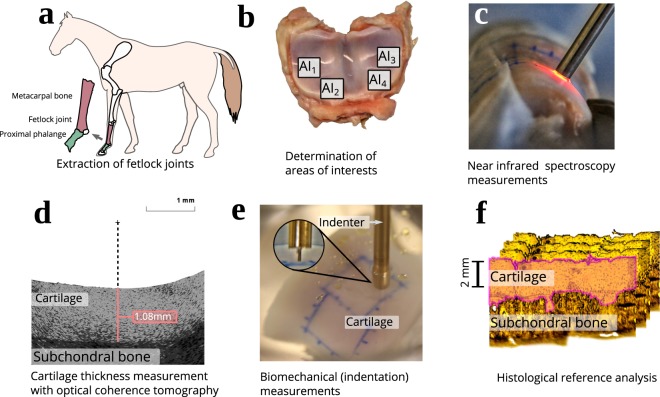
The measurement workflow of the samples. (**a**) Anatomical location of the fetlock joint. (**b**) An example of areas of interest. (**c**) Near infrared spectroscopic measurement. (**d**) Determination of cartilage thickness using optical coherence tomography. (**e**) Material testing setup for conducting the biomechanical indentation tests. (**f**) An example histological section of equine cartilage and subchondral bone.

**Fig. 2:**
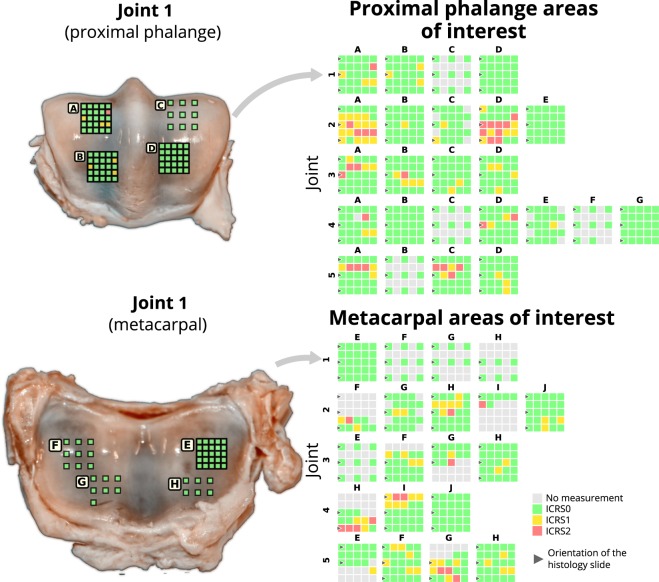
All areas of interest (AI) and associated measurement points with their evaluated cartilage condition (according to ICRS grade scored from optical coherence tomography images)^17^. The locations and size of the AIs are demonstrated on the proximal phalange and metacarpal surfaces of joint 1.

This dataset was collected from five mature equine fetlock joints (Fig. 1a) obtained from a slaughterhouse in Utrecht. Representative areas of interest (AI) with varying degrees of cartilage defects were selected and graded by two experienced veterinary surgeons (Fig. 1b). These regions were first measured with NIRS in a grid-like pattern (Figs 1c and 2). Corresponding reference values for each site were then measured using optical coherence tomography (OCT, Fig. 1d), a biomechanical testing protocol (Fig. 1e), and a battery of histological methods (Fig. 1f). The biomechanical protocol was employed to determine various mechanical properties of the tissue through indentation testing. OCT was used to determine the thickness of the non-calcified cartilage layer at each measurement site. Histological methods (polarized light microscopy (PLM), digital densitometry (DD) of Safranin-O stained samples, and Fourier-transform infrared microspectroscopy (FTIR)) were used as a post-hoc step to determine cartilage collagen fibre orientation, proteoglycan content, and collagen content, respectively, from a selected subset of measurement points. Histological analysis was performed on thin slices cut from the original samples. The collected data was first reported in the studies of Sarin *et al*.^16,17^ and has since been utilized in Prakash *et al*.^18,19^.

We believe the published data can be useful for equine veterinary research or as an animal model of human cartilage research. The provided NIR spectra, in combination with biomechanical indentation testing, can be used to train models capable of predicting various biomechanical properties of cartilage. Likewise, the combination of NIRS with the histological reference parameters can be utilized to predict properties related to the composition and structure of the tissue. The development of new calibration techniques for NIRS is an active field of research and open datasets are used to evaluate the performance of these techniques. In comparison to publicly available datasets^20–27^, the presented data comprises a high number of samples, a large selection of reference variables, and represents various tissue conditions. Currently available NIRS datasets rarely contain measurements of biological tissue but rather focus on agricultural^20–22^, chemical^25^ or food products^26,27^.

While the main focus of the dataset is in the development of NIRS techniques for evaluating cartilage condition, the broad library of reference variables can also be used to study the structure-function relationship of articular cartilage. These functional, compositional, and structural properties could be utilized, for instance, in simulation studies of joint physiology^28^ or simply as a reference library. Finally, the NIRS measurements combined with the structural and compositional properties of cartilage could, for instance, be used to model the interaction between NIR light and articular cartilage in order to gain a better understanding of the sensitivity of the NIRS technique as a function of penetration depth.

## Methods

The following sections (*i*.*e*., *Sample extraction*, *Near infrared spectroscopy*, *Measurement of cartilage thickness*, *Biomechanical testing*, and *Histology*), describing the methods utilized in this study, are expanded versions of descriptions in our related works^16,17^. The employed measurement techniques and the corresponding data are summarized in Table 1.

**Table 1:** Summary of the measurement techniques employed, and corresponding data collected.

Technique	Measurement locations	Data
Near infrared spectroscopy	869	Absorption spectra (λ = 700 nm–1150 nm) with 3 repetitions per measurement location
Optical coherence tomography	869	Cartilage and calcified cartilage thickness
Biomechanics (indentation)	1^st^ protocol, 869	Raw signals (time, location, and load) and calculated parameter: instantaneous modulus
	2^nd^ protocol, 202	Raw signals (time, location, and load) and parameters: equilibrium and dynamic moduli
Digital densitometry	530	Depth-wise optical density profile (average of three histological sections)
Polarized light microscopy	530	Depth-wise collagen orientation and retardance profiles (average of three histological sections)
Fourier transform infrared microspectroscopy	530	Depth-wise proteoglycan and amide profiles (one histological section)

### Sample extraction

Metacarpophalangeal joints (*N* = 5) were extracted from mature equines which were obtained from a slaughterhouse in Utrecht (Equine Slaughterhouse Van de Veen, Nijkerk, Netherlands); no ethical permission was required. A total of 44 AIs (dimensions 15 × 15 mm) with varying cartilage condition were selected from the joints by two experienced equine surgeons. Approximately half of the AIs were selected from the articular surface of the metacarpal bone and the other half from the surface of the proximal phalanx. Each AI was independently scored by the two surgeons according to the International Cartilage Repair Society (ICRS) scoring system. ICRS scores were used to divide the AIs into healthy (*N* = 19) and damaged (*N* = 25) categories. Each individual AI was further subdivided into a uniform 5 × 5 grid (25 measurement points) where NIRS and reference measurements were conducted. In total, 869 measurement points from all AIs were subjected to further analysis, while the remaining 231 points were excluded due to fully eroded cartilage or due to limitations imposed by extensive biomechanical measurements (sample preservation).

### Near infrared spectroscopy

**Fig. 3:**
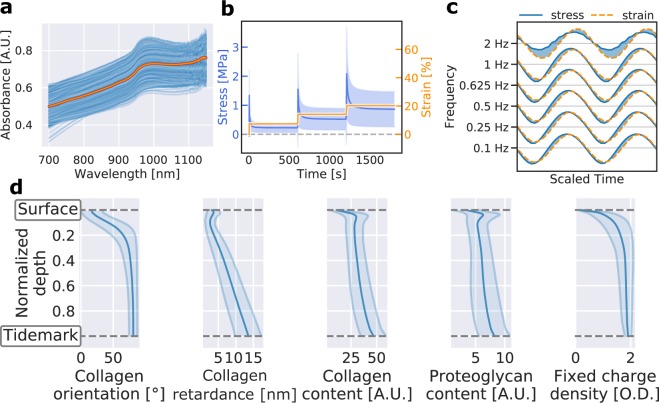
Visualization and technical validation of acquired data. (**a**) All NIR spectra. The thick red line represents the average spectrum. (**b**) Average stress-relaxation curves of cartilage samples. The shaded region corresponds to the standard deviation of stress-values. (**c**) Average stress-strain behaviour per testing frequency in dynamic mechanical testing. Shaded region illustrates the phase difference between stress and strain. (**d**) Averaged depth-wise profiles for collagen network orientation (PLM), retardance (PLM), and content (FTIR) as well as proteoglycan content (FTIR) and fixed charge density (DD). Shaded regions represent the standard deviation.

NIRS measurements were performed using a system consisting of a halogen light source (wavelength 360–2500 nm, power 5 W, optical power 239 µW (in 600 µm fibre), Avantes BV, Apeldoorn, Netherlands), a spectrometer (wavelength 200–1160 nm, AvaSpec-ULS2048XL, Avantes BV), and a diffuse reflectance fibre optic probe^16,17^. The probe (*d* = 5 mm) consists of seven fibres (*d*_*fibre*_ = 600 µm) within the central window (*d* = 2 mm), of which the central fibre was utilized for collecting diffuse reflected light. Data acquisition was performed with Avasoft 8.0 software (Avantes BV). Dark and reference spectra were acquired from non-reflectance (black rubber pad) and reflectance standards (Spectralon, SRS-99, Labsphere Inc., North Sutton, USA), respectively, with the fibre optic probe in perpendicular contact during measurement in order to minimize environmental factors such as stray light. The absorbance at each wavelength (*A*_*λ*_) was determined as follows:

$${A}_{\lambda }=-{log}_{10}\frac{{S}_{\lambda }-{D}_{\lambda }}{{R}_{\lambda }-{D}_{\lambda }},$$

where *S*_*λ*_ is sample spectrum, *D*_*λ*_ is the dark spectrum, and *R*_*λ*_ is the reference spectrum. The absorption spectrum for each measurement location was determined as the average of three consecutive spectral measurements, with each spectrum consisting of eight coadded acquisitions. Data within the spectral region of 700–1050 nm was utilized (Fig. 3a) since light in the visible region penetrates deeper into the tissue and includes strong contributions from the underlying subchondral bone^29,12^. Physiological condition of articular cartilage was preserved by constantly spraying phosphate-buffered saline (PBS) on the sample surface and placing PBS soaked gauze on cartilage surrounding the measurement points. After NIRS, the samples were immersed in PBS and stored at −20 °C until required for reference analyses.

Since spectral data are likely to include hardware-related noise, spectral preprocessing is required to eliminate noise without degrading essential information. The NIR spectra included in this dataset has not been preprocessed in any way, allowing the user to freely choose preprocessing methods they deem necessary. In the original studies of Sarin *et al*., a third-degree Savitzky-Golay filter was utilized for preprocessing prior to analysis. The second derivative spectra were also calculated to remove baseline offset and the dominant linear term from the spectral data^30^. This preprocessing technique was selected as it enhances identification of small and subtle absorption peaks which are not easily resolved visually in the original spectrum^30,31^. Additionally, normalization techniques, such as multiplicative scatter correction and standard normal variate, can be employed to further enhance spectral changes. We have provided an example MATLAB script of a typical analysis which also includes spectral preprocessing (see “Data Records” section).

### Measurement of cartilage thickness

Samples were thawed in PBS at room temperature and subjected to OCT (wavelength 1305 ± 55 nm, axial resolution <20 µm, lateral resolution 25–60 µm; Ilumien PCI Optimization System, St. Jude Medical, St. Paul, MN, USA) to determine non-calcified cartilage thickness without damaging the cartilage (Fig. 1d)^16,17^. The average thickness of equine cartilage was 0.89 mm with a range between 0.32 and 1.82 mm. This information was later required in biomechanical measurements. OCT images were also utilized in the ICRS scoring of cartilage condition^17^.

### Biomechanical testing

The bone end of each sample was glued on a custom-made sample holder which was mounted on a goniometer (#55–841, Edmund Optics Inc., Barrington, NJ, USA)^16,17^. The sample was fully immersed in PBS supplemented with Antibiotic-Antimycotic solution (A5955, Sigma-Aldrich) during measurements (Fig. 1e).

Cartilage biomechanical properties were determined through indentation testing with a custom material tester using plane-ended cylindrical indenters (*d* = 0.53 mm & 0.51 mm). The material tester consisted of a load cell (5 mN resolution, Sensotec, Columbus, OH, USA) and an actuator with a displacement resolution of 0.1 µm (PM500–1 A, Newport, Irvine, CA, USA). Cartilage surface and the indenter were aligned perpendicular, followed by driving the indenter into contact with the surface (pre-stress = 12.5 kPa)^14^. Contact was ensured by indenting the specimen 2% of its thickness five times.

To ensure sample preservation during the extensive biomechanical measurements, two different testing protocols (protocols 1 and 2, see Fig. 3b,c) were used. First, protocol 1, consisting of a single 7.5% strain step indentation at a strain rate of 100%/s, was performed for all measurement points. Second, a more extensive protocol 2 was performed on a select set of measurement locations (five measurement points per AI, *N* = 202). Protocol 2 consisted of an indentation test with three cumulative 7.5% strain steps with 10-minute relaxation time between steps (strain rate 100%/s) followed by four cycles of dynamic sinusoidal loading at frequencies 0.1, 0.25, 0.5, 0.625, 0.833, 1.0, and 2.0 Hz (amplitude of 2% of the remaining cartilage thickness).

Equilibrium, dynamic, and instantaneous moduli were calculated with solution derived from Hayes *et al*.^32^ with Poisson’s ratios of 0.1, 0.5, and 0.5, respectively^33^. Equilibrium modulus was determined from the linear slope of the equilibrium stress-strain curve, whereas dynamic modulus was calculated from sinusoidal loading as the ratio of stress and strain amplitudes. Instantaneous modulus was determined from the first step of the stress-relaxation curves of both protocols

### Histology

Osteochondral samples were processed for histology by extracting the measurement locations (Fig. 2, black arrows), followed by fixing in formalin, decalcification in EDTA, and embedding in paraffin blocks^16,17,34–36^. Sections (*N* = 7) were cut with a microtome for the histological imaging modalities, *i*.*e*., FTIR microspectroscopy (*N* = 1), PLM (*N* = 3), and DD (*N* = 3). The section thicknesses for the imaging modalities were 5 μm, 5 μm, and 3 μm, respectively.

FTIR microspectroscopy was utilized to determine collagen and proteoglycan distributions from the histological sections by mapping 500-μm-wide areas covering the full cartilage thickness in the mid infrared (MIR) region. Similar regions were imaged with PLM and DD. FTIR measurements were conducted with a Thermo iN10 FT-IR microscope (Thermo Nicolet Corporation, Madison, WI, USA) in transmission mode at a spectral resolution of 4 cm^−1^ and pixel size of 25 × 25 μm^2^. Four repetitive measurements per pixel were acquired and averaged. The collagen and proteoglycan contents were determined as the integrated area of the amide I peak (1584–1720 cm^−1^) and the carbohydrate region (984–1140 cm^−1^), respectively^37^.

PLM enabled determination of collagen fibre orientation and birefringence of the cartilage samples. PLM imaging was conducted using an Abrio PLM system (CRi, Inc., Woburn, MA, USA) mounted on a conventional light microscope (Nikon Diaphot TMD, Nikon, Inc., Shinagawa, Tokyo, Japan). The Abrio system consists of a green bandpass filter, a circular polarizer, and a computer-controlled analyser composed of two liquid crystal polarizers and a CCD camera. All specimens were imaged at identical orientation with a 4.0x objective, which resulted in a pixel size of 2.53 × 2.53 μm^2^. In the orientation images, 0 degrees corresponds to the orientation parallel to cartilage surface and 90 degrees perpendicular to cartilage surface.

For DD measurements, the 3 µm thick sections were stained with Safranin-O to determine proteoglycan distribution^26^. The system consists of a light microscope (Nikon Microphot-FXA, Nikon Co., Tokyo, Japan), equipped with a light source, a monochromatic filter, and a 12-bit CCD camera (ORCA-ER, Hamamatsu Photonics K.K., Hamamatsu, Japan). The system was calibrated with neutral density filters (Schott, Mainz, Germany) covering optical density (OD) range from 0 to 3.0. The samples were imaged with a 4.0x objective resulting in a pixel size of 1.56 × 1.56 μm^2^.

## Data Records

The data records consist of four MATLAB (MathWorks Inc., Natick, MA, USA) .mat files housed within figshare^38^. The nirs_and_references.mat within figshare^38^ contains all of the measured NIR spectra and the associated reference values calculated from the biomechanical tests and the histological analysis (see corresponding *Methods*-sections for details on how these values were obtained). This dataset is the most important and practical dataset as it combines the measured signal (*i*.*e*., NIR spectra) and a set of cartilage properties (such as, cartilage thickness, equilibrium modulus, collagen content, etc.). During the calculation of the reference values, several assumptions were made about the data, influencing the final values. For the sake of completeness, transparency, and better replicability, the original data from the reference measurements (with the exception of OCT and PLM) are also included^38^. The ftir_raw.mat within figshare^38^ contains the raw FTIR matrices that were collected from the histological sections as described in the chapter *Histology*. This data was used for determining the proteoglycan and collagen contents as a function of cartilage depth. The biomech_raw_protocol_1.mat and biomech_raw_protocol_2.mat within figshare^38^ contain the measured force and displacement data measured using the biomechanical indentation testing protocols (see *Biomechanical Testing* for details). Each of the .mat files contains a “sample_id” variable, which can be used to link measurements of the same location from different modalities. The motivation for providing raw data was to enable recalculation of the reference variables.

The nirs_and_references.mat contains the NIR spectra and values of the reference parameters which are stored as a MATLAB structure. Each element of the dataset structure corresponds to one measurement point and different fields contain the data. Meta-data, including the joint bone type and AI for each measurement point, is also included. A full list of all the different variables is given in Online-only Table 1: List of variables contained in nirs_and_references.mat.

The ftir_raw.mat contains the raw data matrices of the FTIR microspectroscopy measurements which are also stored as a MATLAB structure. The structure of the dataset is similar to nirs_and_references.mat, where each element of the structure represents a different measurement location. Information about the specific joint and measurement location is encoded in the “sample_id” variable. Variables “wave” and “data” contain the wavenumber vector and the FTIR matrix of that specific point. The FTIR measurement was used to calculate the histological reference values related to proteoglycan and collagen contents.

The biomech_raw_protocol_1.mat contains the raw data from the first biomechanical testing protocol. Information about the measurement location can be found under the “sample_id” variable. Raw data of the indentation testing is stored in the “data” variable and contains the timestamp, position and load of the indenter. The column names of the data-matrix are also stored in the “data_columns” variable. The variable “header” contains measurement-specific information about the test setup.

The biomech_raw_protocol_2.mat contains the raw data from the second biomechanical testing protocol. Each element of the structure corresponds to a biomechanical test conducted at a specific measurement point at a given testing frequency. Measurement point information is stored in the “sample_id” variable and variables “header”, “data”, and “data_columns” are the same as in the biomech_raw_protocol_1.mat. The variable “frequency_hd” corresponds to the frequency at which the dynamic indentation testing was conducted.

## Technical Validation

Dataset size (*N* = 869 or *N* = 530) is sufficient for constructing and validating multivariate models, *e*.*g*., NIRS models. The optimal size of a dataset required to train a multivariate model depends on the application but the general consensus suggests 100 samples as the lower limit^39^.

More importantly, the spread of data should cover the entire natural range of variation found in the mechanical properties of equine cartilage. An earlier investigation of equine proximal phalanx cartilage (*N* = 30) by Brommer *et al*.^40^ reported thickness values of 0.76 ± 0.13 mm, 0.79 ± 0.05 mm, 0.75 ± 0.10 mm, and 0.78 ± 0.11 mm (multiple values reflect various anatomical locations with varying levels of cartilage degeneration). Corresponding values reported for equilibrium modulus were 1.6 ± 0.6 MPa, 1.0 ± 0.4 MPa, 2.8 ± 1.2 MPa, and 2.2 ± 1.1 MPa. By comparing the values for proximal phalanx in this dataset (thickness = 0.84 ± 0.24 mm and equilibrium modulus = 1.98 ± 1.52 MPa), the biomechanical properties are observed to adequately cover the range of values previously reported for this tissue type although the values are acquired from only five individual joints.

Utilization of this dataset for human studies should be reviewed on a case-by-case basis depending on the application of the data. Generally, large mammals, such as equine and bovine, have been considered as suitable animal models for representing human joint physiology due to similarities in loading, gait and cartilage thickness^41^. For equine orthopaedics, the dataset can be directly applied as, for example, racehorses often undergo arthroscopic examinations.

To ensure reproducible NIR measurements, each location was measured three times with the coefficient of variation (CV) of the spectra being 0.82 ± 0.32%^16^. The spectra (Fig. 3a) closely resemble those reported and visualized by Afara *et al*.^42–44^ with the most distinct spectral peak at 950 nm, resulting from second overtones of OH and NH stretching^39,45^.

All histological sections were analyzed via semi-automated software (MATLAB R2016b, MathWorks) in which all sections (DD, PLM, and FTIR) were manually inspected. This inspection ensured that: (1) all locations between modalities were matched, (2) no histological sections contained folded tissue, and (3) that no other mistakes were made during the histological processing. Mammals have a distinct structure of articular cartilage with no substantial differences between the species^46,47^; therefore, the comparison is justifiable. The presented profiles (Fig. 3d) of collagen content, proteoglycan content, and collagen orientation angle closely resemble those previously reported in literature^31,48–51^.

### ISA-Tab metadata file

Download metadata file

## Data Availability

The guidelines and example codes provided in the data description present good practices for preprocessing and analysing spectroscopic (NIR and FTIR) and biomechanical signal. The NIR spectroscopic data is validated through multivariate modelling (*i*.*e*., partial least squares regression) and cross-validation. In addition, the means of calculating the amide (collagen) and proteoglycan images from FTIR images are presented. The custom codes presented were written using MATLAB R2015–2017 (Mathworks Inc., Natick, MA, USA). Examples codes are available in a public repository (https://github.com/uef-bbc/sarin-scientific-data-2019).

## Online-only Table

**Online-only Table 1:** List of variables contained in nirs_and_references.mat.

Variable name	Type	Unit	Description
plm_retardance_profile	vector	nm	PLM retardance profile as a function of cartilage depth.
plm_orientation profile	vector	deg.	PLM profile as a function of cartilage depth.
ftir_proteoglycan_profile	vector	A.U.	FTIR proteoglycan content profile as a function of cartilage depth.
ftir_collagen_profile	vector	A.U.	FTIR collagen content profile as a function of cartilage depth.
dd_profile	vector	O.D.	Depth-wise DD profile of the cartilage cross-section.
dd_average_surface	scalar	O.D.	Average DD value in superficial cartilage.
dd_average_deep	scalar	O.D.	Average DD value in deep cartilage.
plm_average_surface	scalar	deg.	Average PLM value in superficial cartilage.
plm_average_deep	scalar	deg.	Average PLM value in deep cartilage.
ftir_collagen_average_surface	scalar	A.U.	Average collagen content in superficial cartilage (computed from FTIR).
ftir_collagen_average_deep	scalar	A.U.	Average collagen content in deep cartilage (computed from FTIR).
ftir_proteoglycan_average_surface	scalar	A.U.	Average proteoglycan content in superficial cartilage (computed from FTIR).
ftir_proteoglycan_average_deep	scalar	A.U.	Average proteoglycan content in deep cartilage (computed from FTIR).
ftir_collagen_2d	matrix	A.U.	2D cross-section FTIR matrix of collagen content (width x depth)
ftir_proteoglycan_2d	matrix	A.U.	2D cross-section FTIR matrix of proteoglycan content (width x depth)
wavelength	vector	nm	Wavelength vector of the NIR spectra.
sample_id	string	-	Unique identifier string for this measurement.
joint_id	integer	-	Identifier of the fetlock joint.
area_of_interest_id	string	-	Identifier for the area of interest.
measurement_point_id	integer	-	Identifier for the measurement point inside area of interest.
bone_type	string	-	Type of bone surface (metacarpal or proximal phalanx)
raw_spectra	matrix	A.U.	Matrix containing three NIR spectra measured from this location.
cartilage_thickness	scalar	mm	Thickness of the articular cartilage layer.
calcified_layer_thickness	scalar	mm	Cartilage thickness including calcified layer.
indenter_diameter	scalar	mm	Diameter of the indenter used for mechanical testing.
instantaneous_modulus	scalar	MPa	Value of cartilage instantaneous modulus.
equilibrium_modulus	scalar	MPa	Value of cartilage equilibrium modulus.
dynamic_modulus_at_01 hz	scalar	MPa	Value of cartilage dynamic modulus at 0.1 Hz loading.
dynamic_modulus_at_025 hz	scalar	MPa	Value of cartilage dynamic modulus at 0.25 Hz loading.
dynamic_modulus_at_05 hz	scalar	MPa	Value of cartilage dynamic modulus at 0.5 Hz loading.
dynamic_modulus_at_0625hz	scalar	MPa	Value of cartilage dynamic modulus at 0.625 Hz loading.
dynamic_modulus_at_0833 hz	scalar	MPa	Value of cartilage dynamic modulus at 0.833 Hz loading.
dynamic_modulus_at_1 hz	scalar	MPa	Value of cartilage dynamic modulus at 1 Hz loading.
dynamic_modulus_at_2 hz	scalar	MPa	Value of cartilage dynamic modulus at 2 Hz loading.
